# A Ketogenic Diet Improves Cognition and Has Biochemical Effects in Prefrontal Cortex That Are Dissociable From Hippocampus

**DOI:** 10.3389/fnagi.2018.00391

**Published:** 2018-12-03

**Authors:** Abbi R. Hernandez, Caesar M. Hernandez, Keila Campos, Leah Truckenbrod, Quinten Federico, Brianna Moon, Joseph A. McQuail, Andrew P. Maurer, Jennifer L. Bizon, Sara N. Burke

**Affiliations:** ^1^Department of Neuroscience, McKnight Brain Institute, University of Florida, Gainesville, FL, United States; ^2^Institute on Aging, University of Florida, Gainesville, FL, United States

**Keywords:** anxiety, GABA, glucose, glutamate, metabolism, monocarboxylate, transporter

## Abstract

Age-related cognitive decline has been linked to a diverse set of neurobiological mechanisms, including bidirectional changes in proteins critical for neuron function. Importantly, these alterations are not uniform across the brain. For example, the hippocampus (HPC) and prefrontal cortex (PFC) show distinct patterns of dysfunction in advanced age. Because higher cognitive functions require large–scale interactions across prefrontal cortical and hippocampal networks, selectively targeting an alteration within one region may not broadly restore function to improve cognition. One mechanism for decline that the PFC and HPC share, however, is a reduced ability to utilize glucose for energy metabolism. Although this suggests that therapeutic strategies bypassing the need for neuronal glycolysis may be beneficial for treating cognitive aging, this approach has not been empirically tested. Thus, the current study used a ketogenic diet (KD) as a global metabolic strategy for improving brain function in young and aged rats. After 12 weeks, rats were trained to perform a spatial alternation task through an asymmetrical maze, in which one arm was closed and the other was open. Both young and aged KD-fed rats showed resilience against the anxiogenic open arm, training to alternation criterion performance faster than control animals. Following alternation testing, rats were trained to perform a cognitive dual task that required working memory while simultaneously performing a bi-conditional association task (WM/BAT), which requires PFC–HPC interactions. All KD-fed rats also demonstrated improved performance on WM/BAT. At the completion of behavioral testing, tissue punches were collected from the PFC for biochemical analysis. KD-fed rats had biochemical alterations within PFC that were dissociable from previous results in the HPC. Specifically, MCT1 and MCT4, which transport ketone bodies, were significantly increased in KD-fed rats compared to controls. GLUT1, which transports glucose across the blood brain barrier, was decreased in KD-fed rats. Contrary to previous observations within the HPC, the vesicular glutamate transporter (VGLUT1) did not change with age or diet within the PFC. The vesicular GABA transporter (VGAT), however, was increased within PFC similar to HPC. These data suggest that KDs could be optimal for enhancing large-scale network function that is critical for higher cognition.

## Introduction

Advanced agemultiple is associated with declines across cognitive domains, including episodic memory (Grady et al., 1999; Harada et al., 2013) and executive functions (Bizon et al., 2012; Bañuelos et al., 2014; Hernandez et al., 2017c), as well as an increased prevalence of anxiety disorders (Bryant et al., 2008). Critically, different cognitive functions do not decline in isolation and an enhanced vulnerability to anxiety can feedforward to impair memory and executive functions (Wetherell et al., 2002; Whyche, 2008; Dissanayaka et al., 2017). Thus, finding therapeutic strategies that can confer resilience to anxiety and other cognitive impairments associated with aging is critical for promoting the ability of older adults to live independently.

Different brain regions show distinct patterns of neurobiological alterations in aging, presenting a critical barrier to the development of therapeutics that effectively mitigate decline of cognitive functions that depend on inter-connected neural structures, including the prefrontal cortex (PFC) and hippocampus (HPC; reviewed in Burke and Barnes, 2006; McQuail et al., 2015). Thus, restoring function in one area may exacerbate decline in another, failing to restore behavioral output. In fact, the PFC and HPC are both neural hubs in large-scale networks that support cognition and are known to have distinct patterns of dysfunction in old animals. For example, within the CA3 subregion of the HPC there is hyperactivity (Wilson, 2005; Yassa et al., 2011; Thomé et al., 2015; Maurer et al., 2017). In contrast, for the PFC, biochemical (Bañuelos et al., 2014) and electrophysiological (Carpenter et al., 2016) data suggest that inhibitory tone is increased. This disrupted balance between excitation and inhibition (for review see McQuail et al., 2015) may impede the appropriate engagement of PFC circuitry during complex behaviors (Cabeza et al., 1997; Grady et al., 1999; Nagel et al., 2009; Cappell et al., 2010), leading to a reduction in the dynamic range of PFC activity.

Not surprisingly, because functional interactions between the PFC and medial temporal lobe are essential for higher cognitive function, behaviors that rely on communication between these brain regions appear particularly vulnerable in advanced aged. Specifically, the ability to multi-task by using working memory while simultaneously performing a bi-conditional association task (WM/BAT), which requires communication between the PFC and medial temporal lobe (Jo and Lee, 2010; Hernandez et al., 2017b), shows age-related declines in performance prior to observable impairments on the HPC-dependent Morris watermaze test of spatial reference memory (Hernandez et al., 2015). Moreover, age-related changes in neuron activity patterns during WM/BAT performance are most evident in the subset of medial temporal lobe cells that project directly to PFC (Hernandez et al., 2018b) These findings corroborate the idea that behaviors relying on PFC–HPC interactions are particularly sensitive to decline during advancing age.

While the PFC and HPC show distinct mechanisms of age-related dysfunction, one variable that appears to be ubiquitous across regions is a declining ability to utilize glucose as an energy substrate. Notably, glucose metabolism within the brain is significantly lower in aged study participants (Petit-Taboué et al., 1998; Gold, 2005; Rasgon et al., 2005) and this decline correlates with impaired behavior on the HPC-dependent Morris watermaze task and a spatial alternation task (Gage et al., 1984; Gold, 2005). Furthermore, cognitively impaired aged rats have larger decreases in extracellular HPC glucose levels during task training relative to those observed in young animals (Gold, 2005). In humans there is an age-related decrease in brain glucose uptake that exceeds that of oxygen use, resulting in loss of brain aerobic glycolysis (Goyal et al., 2017). These metabolic deficits in older adults are a likely contributor to cognitive impairments, as the ability to produce ATP declines by 8% per decade of life (Short et al., 2005), making aged individuals particularly vulnerable to metabolic alterations. Moreover, metabolic syndrome (Agrawal and Gomez-Pinilla, 2012) and insulin insensitivity are associated with cognitive deficits (for review, see Greenwood and Winocur, 2005). Together, these data indicate the strong association between metabolism and cognitive function.

While over consumption of an obesogenic high fat and high sugar diet leads to loss of dendritic spines in the PFC and perirhinal cortex, as well as to impairments in spontaneous object recognition tasks and set-shifting (Bocarsly et al., 2015), to date, very few studies have examined the ability of diet to enhance cognition and improve synaptic function. Ketogenic diets (KDs) are high in fat and low in carbohydrates, which leads to the production of fat-derived ketone bodies within the liver that enter the Krebs cycle for ATP production (Krebs, 1966; Egan and D’Agostino, 2016). The ability of these diets to bypass glucose/insulin signaling, which is compromised in old animals, suggests a potential utility in enhancing brain function across the lifespan. Previously, we have shown that 12 weeks of a KD can alter the expression of glucose and monocarboxylate transporters in the HPC, and reinstate expression of the vesicular glutamate transporter in old animals to the levels observed in young (Hernandez et al., 2018a).

The current study examined the extent to which cognitive performance, membrane transporters and markers of synaptic function could be altered by a KD in young and aged rats. Animals were placed on a nutrient- and calorie-matched KD or control diet (CD) for 12 weeks (described in detail in Hernandez et al., 2018a) prior to testing on tasks that included an elevated figure-8 spatial alternation task, which required animals to overcome anxiety about being in open areas to obtain a reward, and the WM/BAT that test cognitive multi-tasking abilities. The elevated figure-8 spatial alternation task used here has advantages, relative to the standard elevated plus maze test of anxiety-like behaviors, as animals are trained to ambulate through the maze for a reward. Thus, their natural habituation is not associated with a decrease in exploratory behavior and this task can be tested daily to measure the extent to which animals are able to overcome anxiety regarding open spaces to retrieve a reward. Importantly, because affective behaviors (Adhikari et al., 2010; Godsil et al., 2013) and WM/BAT performance (Lee and Solivan, 2008; Jo and Lee, 2010; Kim et al., 2011; Hernandez et al., 2017b, 2018b) both involve HPC- PFC circuitry, we also examined PFC expression of transporters previously shown to be affected by the KD in the HPC (Hernandez et al., 2018a).

## Materials and Methods

### Subjects and Handling

Young (4 months; *n* = 16 male, *n* = 2 female) and aged (20 months; *n* = 18 male, *n* = 3 female) Fischer 344 × Brown Norway F1 Hybrid rats from the NIA colony at Taconic Farms were used in the behavioral study. While the importance of the inclusion of both sexes is recognized, limited availability of female rats from the NIA colony precluded the use of equal numbers of male and female rats in this study. There were 8 young males, 1 young female, 8 aged males and 1 aged female rats in the KD group and there were 8 young males, 1 young female, 10 aged males and 2 aged female rats in the CD group. Importantly, data from the female rats fell within the range of the males. Tissue from an additional 12 young and 12 aged male Fischer 344 × Brown Norway F1 Hybrid rats was used for the transporter quantification, split evenly among the two diet groups. These rats were used in a previous study utilizing the same KD implemented here (Hernandez et al., 2018a). Rats were housed individually and maintained on a 12-hr light/dark cycle, and all behavioral testing was performed in the dark phase. Rats were given 1 week to acclimate to the facility prior to blood collection. All experimental procedures were performed in accordance with National Institutes of Health guidelines and were approved by Institutional Animal Care and Use Committees at the University of Florida.

### Diet

Prior to diet administration, rats were randomly assigned to either a high-fat, low-carbohydrate KD (75.85% fat, 20.12% protein, 3.85% carbohydrate; Lab Supply; 5722, Fort Worth, Texas) mixed with MCT oil (Neobee 895, Stephan, Northfield, Illinois) or a calorically and micronutrient matched CD (CD; 16.35% fat, 18.76% protein, 64.89% carbohydrate; Lab Supply; 1810727, Fort Worth, Texas; for details on diet, see Hernandez et al., 2018a). Rats were weighed daily and given ∼51 kcal at the same time each day for the first 12 weeks of the diet. This caloric quantity, which is sufficient for healthy weight maintenance on both a ketogenic and CD (Hernandez et al., 2017a), was given to ensure rats were not eating variable amounts of calories across groups, and thus energy intake could not account for variable behavioral differences. For the rats participating in the behavioral assay, following a 12-week adjustment period to the diet and confirmation of sustained nutritional ketosis, the amount of food was restricted to cause an ∼15% reduction in body weight to motivate participation during the behavioral assay. Access to water was *ad libitum*.

### Confirmation of Ketosis

β-hydroxybutyrate (BHB; mmol; L), the prominent ketone body in the blood, and glucose levels (mg; dL) were determined using the Precision Xtra blood monitoring system (Abbott Diabetes Care, Inc., Alameda, CA, United States). Rats were briefly restrained and received a small nick to the tail using a sterile scalpel blade to obtain blood for monitoring glucose and ketone body levels. Measurements were taken 1 h after eating each week for the first 3 weeks of dietary intervention. Following confirmation of nutritional ketosis throughout the first 3 weeks, weekly blood draws were stopped to prevent undue stress to the animals. However, intermittent testing continued between weeks 4–29 to ensure sustained ketosis.

### Behavioral Testing Apparatus

**FIGURE 1:**
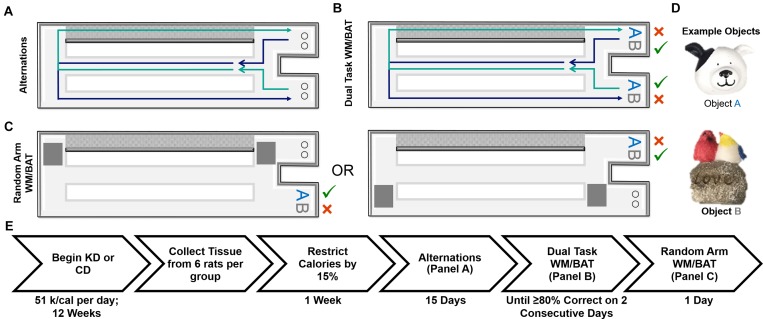
Schematic of behavioral tasks utilized and experiment timeline. **(A)** Elevated figure-8 shaped maze used for all experimental testing in which rats were first trained to alternate between the left and right arms. Note the right arm was enclosed on both sides by white walls (gray for depiction purposes only) and the left arm was open on both sides. **(B)** Dual task WM/BAT took place using one object pair, but the correct object was contingent on location within the maze. **(C)** This same maze was used for random arm BAT, with the same objects, but one arm was randomly blocked off each trial by the experimenter. **(D)** Example of objects used for WM/BAT testing. **(E)** Timeline of diet implementation and behavioral testing.

All behavioral testing occurred on a figure 8-shaped maze (see Figure 1) 67.5 inches long and 25 inches wide. The maze was constructed from wood and sealed with waterproof white paint. The center arm was made of clear acrylic. The choice platforms each contained two food wells (2.5 cm in diameter) that were recessed into the maze floor by 1 cm. All arms were 4 inches wide. The choice platforms were contained within 7.5 cm raised walls and the right arm was contained within 20 cm high raised halls, but the center and left arms did not have walls. Thus, the arms of the maze had an asymmetry such that only the right arm was enclosed while the animals were relatively more exposed on the middle and left arms. To dampen the influence of extraneous noise on behavior, a white noise machine was used during behavioral training and testing.

### Behavioral Habituation, Shaping, and Spatial Alternation

Rats were placed on the maze for 10 min per day for two consecutive days to habituate them to the maze. Food rewards (macadamia nuts, Mauna Loa, Keaau, Hawaii) were scattered throughout the maze to encourage exploration. Macadamia nuts, used for rats on both diets, were chosen based on their palatability and macronutrient composition (80% fat, 13.33% carbohydrates, and 6.67% protein), which would not inhibit ketosis. Importantly, the macadamia nuts did not interfere with the consumption of the CD or lead to weight gain. Rats were then trained to alternate between the two outside arms of the maze in a figure-8 motion, returning through the center between trials (see Figure 1A). Rats were considered to be successfully alternating when they went the correct direction on at least 16 of 20 trials on two consecutive days. For a complete timeline of each behavioral assay, see Figure 1E.

### Dual Working Memory/Bi-Conditional Association Task (WM/BAT)

Following alternation training, rats were trained to perform simple object discriminations both with and without alternations. These tasks did not have the bi-conditional object-in-place rule. Similar to previous reports that have shown no effect of age on the ability to discriminate between objects that do not share feature overlap (Burke et al., 2011; Hernandez et al., 2015; Johnson et al., 2017), there were no effects of age or diet (*p* ≥ 0.29 for all effects and interactions; data not shown). Rats were then tested on the cognitive dual task which combined spatial alternations with a bi-conditional asssociation task (WM/BAT) that required the animal to continue to alternate, while also associating a particular object of a pair with the different testing platforms (see Figure 1B). Specifically, rats are presented with the same two objects (see Figure 1D for an example object pair) in both arms with one object being rewarded in one arm and the alternate object being rewarded in the other arm, regardless of which well they were covering within the choice platform. On the first day of testing, objects were only partially covering the food reward for the first four trails per object (8 trials total) to encourage learning. Rats could begin with a trial toward either arm, but on all subsequent trials, rats had to alternate to the opposite arm utilized in the previous trial. Should a rat mistakenly enter the wrong arm (for example, entering into the right arm following a right arm trial), the trial was recorded as a working memory error and rats were not presented with the object discrimination problem. Rats were tested on this paradigm for fifteen consecutive days. A small subset of rats (*n* = 6 young; 7 aged), needed to be retested on day 13 due to technical difficulties, this occurred in rats on the KD and CDs and impacted the diet groups similarly.

### Random Arm Bi-Conditional Association Task (BAT)

To ensure rats were not adopting an every-other-trial alternation strategy with the objects rather than using spatial information to determine the correct choice, rats were randomly forced into a single arm with left versus right arms randomly varying across trials (random arm BAT), thus randomizing the order in which rats were presented with object A correct trials and object B correct trials. One arm of the maze was blocked off by a second familiar experimenter as rats approached the decision platform, thus ensuring they entered the correct arm of the maze. Rats were given 20 trials on 1 day of testing using the same objects used for WM/BAT.

### Preparation of Tissue and Western Blotting

The non-behaviorally characterized rats (used in Hernandez et al., 2018a) were sacrificed following 12 weeks on the diet and tissue was collected to evaluate transporter expression within the medial PFC. Rats were placed into a bell jar containing isoflurane-saturated cotton (Abbott Laboratories, Chicago, IL, United States), separated from the animal by a wire mesh shield. Animals lost righting reflex within 30 s of being placed within the jar and immediately euthanized by rapid decapitation. Tissue was extracted and immediately frozen on dry ice and stored at -80°C until use. Prefrontal cortical tissue was kept frozen while sliced at 200 μm and tissue punches were taken from the infralimbic, prelimbic, and anterior cingulate regions and homogenized together to get one PFC sample per rat, as each region alone is insufficient for protein analysis of this quantity. Previous studies utilizing this methodology were able to correlate behavioral outcomes with changes in protein within the PFC (Beas et al., 2017; Hernandez et al., 2018c). The membrane and soluble fractions were isolated according to previously published procedures (McQuail et al., 2012; Hernandez et al., 2018a). 5 μg of protein; lane were separated on 4–15% TGX gels (Bio-Rad Laboratories, Hercules, CA, United States) at 200 V for 40 min in tris-glycine running buffer (Bio-Rad). Total protein was transferred to a 0.45 μm pore nitrocellulose membrane at 20 V for 7 min using iBlot Gel Transfer Nitrocellulose Stacks (NR13046-01, Invitrogen, Waltham, MA, United States) and an iBlot machine (Invitrogen, Waltham, MA, United States). All experiments were conducted in triplicate, and the loading order of samples was randomized between gels and experiments to control for systematic variation in the electrophoresis and electroblotting procedures.

Immediately after transfer, membranes were stained for total protein using LiCor’s Revert total protein stain for 5 min (Li-Cor, 926-11011) and scanned using a 685 nm laser on an Odyssey IR Scanner (Li-Cor, Lincoln, Nebraska United States) to detect total protein levels. There were no significant differences in the amount of total protein detected across groups. Membranes were then placed into Rockland blocking buffer (Rockland Blocking Buffer, Rockland, Gilbertsville, PA, United States) for 1 h at room temperature. After blocking membranes were incubated at 4°C overnight with antibodies raised against MCT1, MCT2, and MCT4 (ketone body, lactate and pyruvate transporters); GLUT1, GLUT3 (glucose transporters); VGLUT1 (vesicular glutamate transporter 1), or VGAT [vesicular GABA transporter (VGAT); see Hernandez et al., 2018a]. Membranes were washed in tris buffered saline before incubation in donkey anti-rabbit or donkey anti-mouse secondary antibodies conjugated to IRDye800 (diluted 1:15000; LI-COR). Blots were scanned using a 785 nm laser on an Odyssey IR Scanner. All banding patterns observed were consistent with previous findings (Bañuelos et al., 2014; Beas et al., 2017; Hernandez et al., 2018a). The target protein:total protein ratio was calculated for each technical replicate and data from each independent biological sample were transformed to percent expression of the young, CD-fed group (i.e., mean of this group is set to 100%) for statistical analysis.

### Statistical Analyses

BHB, glucose levels, percent of correct turns, and percent of correct object choices for each task were analyzed using a two-factor ANOVA with the between subjects factors of age (2 levels: young and aged) and diet (2 levels: control and ketogenic). Tasks in which the percent of correct object choices or correct alternation choices were compared across multiple days or multiple arms were analyzed using repeated measures-ANOVAs (RM-ANOVA) with the between subjects factors of diet and age. Side bias indices were calculated for each rat for each day of training as (total rightward turns/total turns). A score of 0.5 indicates turning left and right equally, scores >0.5 indicate a rightward bias and a score <0.5 indicates a leftward bias. All analyses were performed with Statistical Package for the Social Sciences (SPSS) v22. Statistical significance was considered at *p*-values less than 0.05.

## Results

### Confirmation of Nutritional Ketosis

Nutritional ketosis for the 24 rats used in the transporter quantification assay was verified and the results were previously published (Hernandez et al., 2018a), thus this analysis was restricted to animals used in the behavioral assay. Prior to dietary intervention, there was not a significant effect of age [*F*_(1,35)_ = 3.90; *p* = 0.06] or diet group [*F*_(1,35)_ = 0.49; *p* = 0.49] on β-hydroxybutyrate (BHB) levels within the blood, nor was there a significant interaction between the two [*F*_(1,35)_ = 0.00; *p* = 0.95]. At baseline, glucose levels were also not significantly different between age [*F*_(1,35)_ = 0.04; *p* = 0.84] and diet groups [*F*_(1,35)_ = 0.23; *p* = 0.63], nor was the interaction between age and diet group significantly different [*F*_(1,35)_ = 3.06; *p* = 0.09; see Table 1 for raw values]. A repeated measures ANOVA (RM-ANOVA) on BHB levels for weeks 0, 1, 3, and 22 revealed a significant main effect of day [*F*_(3,105)_ = 35.85; *p* < 0.001]. There was a significant effect of diet on BHB levels [*F*_(1,35)_ = 294.12; *p* < 0.001], but not age [*F*_(1,35)_ = 2.93; *p* = 0.10], nor did these two variables significantly interact [*F*_(1,35)_ = 1.29; *p* = 0.26]. The day of testing also significantly interacted with diet group [*F*_(3,105)_ = 49.59; *p* < 0.001], but not with age [*F*_(3,105)_ = 0.75; *p* = 0.52]. A similar repeated measures ANOVA (RM-ANOVA) on glucose levels for weeks 0, 1, 3, and 22 revealed a significant main effect of day [*F*_(3,105)_ = 22.33; *p* < 0.001]. There was a significant effect of diet on glucose levels [*F*_(1,35)_ = 59.57; *p* < 0.001], but not age [*F*_(1,35)_ < 0.01; *p* = 0.97), nor did these two variables significantly interact [*F*_(1,35)_ = 1.60; *p* = 0.21]. The day of testing also significantly interacted with diet group [*F*_(3,105)_ = 25.47; *p* < 0.001], but not with age [*F*_(3,105)_ = 1.20; *p* = 0.31].

**Table 1:** Glucose and β-hydroxybutyrate (BHB) levels before and during dietary implementation. All values are group means ± the standard error of the mean (SEM).

		Before	Week 1	Week 2	Week 3	Week 12	Final
Young control	Glucose (mg/dL)	93.19 ± 4.94	83.31 ± 4.04	97.56 ± 6.26	93.38 ± 2.64	96.63 ± 2.40	108.00 ± 7.19
	BHB (mmol/L)	0.72 ± 0.04	0.63 ± 0.03	0.57 ± 0.04	0.54 ± 0.03	0.59 ± 0.04	0.88 ± 0.08
Young keto	Glucose (mg/dL)	85.21 ± 3.01	63.22 ± 3.84	54.66 ± 4.54	57.63 ± 4.82	62.50 ± 4.04	79.36 ± 4.88
	BHB (mmol/L)	0.75 ± 0.05	2.40 ± 0.22	3.45 ± 0.34	3.59 ± 0.40	3.36 ± 0.42	3.31 ± 0.36
Aged control	Glucose (mg/dL)	98.11 ± 8.30	99.68 ± 6.67	93.32 ± 4.21	100.67 ± 5.01	104.16 ± 4.79	102.75 ± 7.66
	BHB (mmol/L)	0.66 ± 0.03	0.56 ± 0.03	0.53 ± 0.04	0.49 ± 0.02	0.57 ± 0.03	0.76 ± 0.07
Aged keto	Glucose (mg/dL)	100.56 ± 3.28	81.93 ± 8.41	71.44 ± 10.31	68.25 ± 7.36	69.19 ± 3.17	73.07 ± 4.23
	BHB (mmol/L)	0.63 ± 0.04	1.91 ± 0.19	2.81 ± 0.28	2.64 ± 0.25	3.04 ± 0.24	3.37 ± 0.32

**FIGURE 2:**
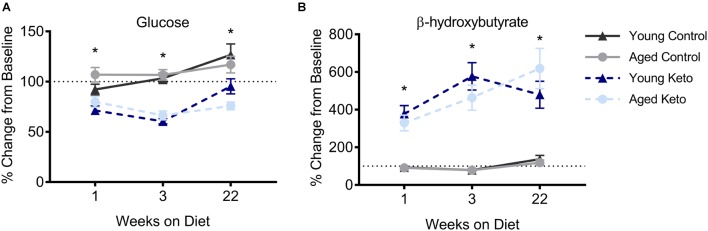
Confirmation of nutritional ketosis following administration of a ketogenic diet in animals used for the behavioral assay. **(A)** Glucose levels significantly differed across diet groups, but not across age groups, throughout the duration of the diet. **(B)** Similarly, β-hydroxybutyrate (BHB) levels were significantly elevated in rats on the KD relative to rats on the CD, but did not differ across age groups, throughout the duration of the diet. All values represent the mean ± SEM, ^∗^indicates main effect of diet.

For each successive time point, the glucose and BHB values were divided by the pre-diet values and multiplied by 100 to determine the percent of baseline. Following 1 week of dietary intervention, a significant main effect of diet group on both BHB [*F*_(1,35)_ = 76.25; *p* < 0.001] and glucose [*F*_(1,35)_ = 38.19; *p* < 0.001] levels was detected, although there was no main effect of age (*p* > 0.73 for both) nor interaction between age and diet (*p* > 0.85 for both; Figure 2). BHB and glucose levels were tested again after 3 weeks on the diet. A similar pattern was observed such that there was main effect of diet group on BHB [*F*_(1,35)_ = 81.70; *p* < 0.001] and glucose levels [*F*_(1,35)_ = 84.06; *p* < 0.001], but no main effect of age (*p* > 0.38 for both), nor a significant interaction between the two (*p* > 0.43 for both). The main effect of diet on BHB and glucose persisted throughout the duration of the experiment and was still present during week 22 of the diet [BHB: *F*_(1,35)_ = 76.59; *p* < 0.001; glucose: *F*_(1,35)_ = 39.74; *p* < 0.001] with no effect of age (*p* > 0.22 for both) nor significant interaction effect between age and glucose or BHB levels (*p* > 0.30 for both). Thus, all rats on the KD had significantly elevated BHB levels and significantly reduced blood glucose levels throughout the duration of behavioral testing.

### KD-Fed Rats Required Fewer Training Sessions to Alternate, Despite Required Entry Into an Anxiolytic Open Arm, Than CD-Fed Rats

KDs have been shown to reduce anxiety (Kashiwaya et al., 2013; Ari et al., 2016; Bostock et al., 2017). Therefore, subjects were trained to on an elevated figure-8 spatial alternation task. This task requires rats to alternate between a closed right arm and a left open arm for 20 trials per day until they were able to maintain ≥80% correct choices on two consecutive days (see Figure 1A). It is well established that rodents prefer to be in the enclosed arms of a maze, rather than open arms, and time spent avoiding an open arm is a measure of anxiety-like behavior in rodents (Pellow and File, 1986; Rodgers and Dalvi, 1997; Bailey and Crawley, 2009). In the elevated figure-8 spatial alternation task this leads to a turn bias toward the closed arm rather than to alternation behavior. Thus, to successfully alternate, rats had to overcome their anxiety about being in open areas to obtain a reward.

**FIGURE 3:**
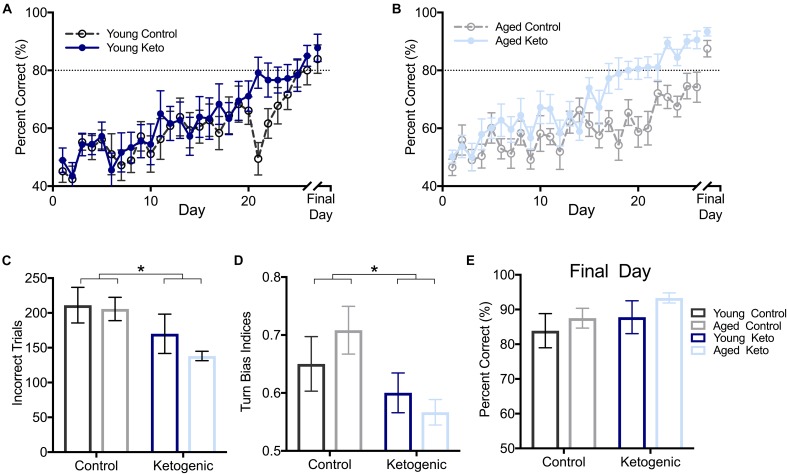
Performance by age and diet on ability to alternate within the elevated figure-8 maze. **(A)** Percent correct alternations during training for young rats within each diet group. **(B)** Percent correct alternations during training for aged rats within each diet group. **(C)** Number of incorrect trials before reaching criterion performance (≥80% correct turns) revealed that there was no significant main effect of age (*p* = 0.37), but there was a significant main effect of diet (*p* = 0.01). **(D)** Similarly, there was a main effect of diet (*p* = 0.02) but not age (*p* = 0.75) on the turn bias on day 20 of testing, as KD-fed rats were able to overcome their closed side bias by this day while CD-fed rats were not. **(E)** Performance on the final day of alternation training for all rats indicated all rats were able to perform similarly on alternations prior to moving on to subsequent tasks regardless of age and diet (*p* > 0.14 for both). All values represent the mean ± SEM; ^∗^ indicates main effect of diet.

As expected, all rats were biased to turn to the right closed arm throughout testing, as indicated by a turn bias > 0.50. A repeated measures ANOVA (RM-ANOVA) on percent correct for each day of training revealed a significant main effect of day [*F*_(1,25)_ = 17.73; *p* < 0.001] but no effect of age [*F*_(1,25)_ = 2.14; *p* = 0.15]. Irrespective of age, there was a significant main effect of diet [*F*_(1,29)_ = 8.75; *p* = 0.006], as well as an interaction between diet and day of training [*F*_(1,29)_ = 1.82; *p* = 0.008; Figures 3A,B]. Furthermore, the number of incorrect trials performed until criterion performance was achieved was compared across groups and there was a significant main effect of diet [*F*_(1,35)_ = 6.86; *p* = 0.01; Figure 3C] indicating that the KD-fed group required significantly fewer trials to reach criterion than the CD-fed group. There was no significant effect of age [*F*_(1,35)_ = 0.81; *p* = 0.37] or interaction between age and diet [*F*_(1,35)_ = 0.41; *p* = 0.53] on number of incorrect trials to criterion.

To determine if there was a pattern to the errors made during the alternation task indicative of rats avoiding the open arm, a turn bias index was calculated for each day of training (total rightward turns/total turns). The first day on which any group reached criterion performance was the aged KD group on day 20 of training (80.56 ± 3.67). On this day, there was a significant effect of diet [*F*_(1,35)_ = 6.12; *p* = 0.02] on side bias, but there was not an effect of age [*F*_(1,35)_ = 0.10; *p* = 0.75] nor was the interaction between age and diet significant [*F*_(1,35)_ = 1.40; *p* = 0.25; Figure 3D]. Interestingly, KD-fed rats did not have a turn bias in either direction, while CD rats still had a rightward (closed arm) turn bias, indicating KD rats were able to overcome their rightward bias earlier in training.

Rats were continuously trained on the alternation task until criterion performance was reached and on the final day of testing for each rat, there were no differences across age [*F*_(1,35)_ = 1.01; *p* = 0.32] or diet [*F*_(1,35)_ = 2.33; *p* = 0.14] groups on their ability to alternate prior to moving on to the WM/BAT testing (Figure 3E). Thus, while KD rats were faster to acquire the correct alternation strategy, all rats were eventually able to alternate at a score of ≥80% correct alternations before the addition of object choices.

### KD-Fed Rats Acquired the Object-in-Place Rule More Quickly Than CD-Fed Rats

**FIGURE 4:**
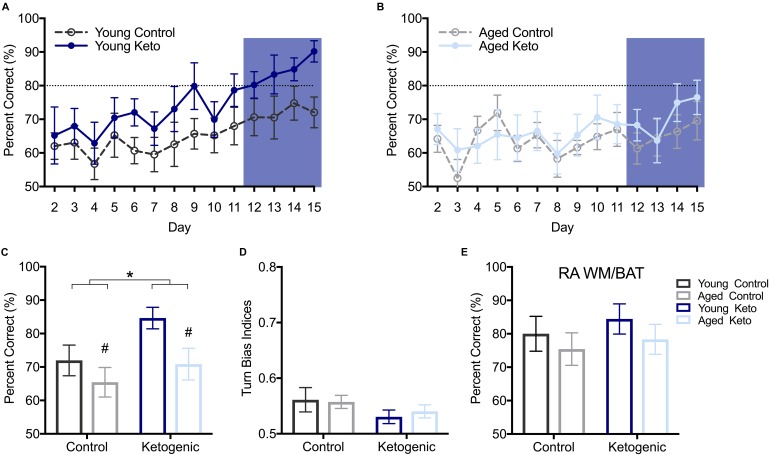
Performance on the working memory/bi-conditional association tasks (WM/BAT). **(A)** Percent correct object choices (*y*-axis) on each day of WM/BAT training (*x*-axis) in young rats within each diet group. **(B)** Percent correct object choices (*y*-axis) on each day of WM/BAT training (*x*-axis) in aged rats within each diet group. The first day on which any group reached criterion performance was day 12, thus performance across days 12–15 were analyzed (blue box). **(C)** There was a significant main effect of both diet (*p* < 0.05) and age (*p* = 0.03) on WM/BAT performance on days 12–15. **(D)** There was no effect of either age or diet on turn bias during days 12–15 (*p* ≥ 0.12 for both). **(E)** Performance on random arm BAT (RA BAT) was not affected by either age or diet (*p* ≥ 0.28 for both), nor did it differ from performance on regular WM/BAT on the final day of testing (*p* 0.22). All values represent the mean ± SEM; #indicates main effect of age, ^∗^indicates main effect of diet.

After the alternation task, rats were trained on WM/BAT in the elevated figure-8 maze for 15 days (see Figure 1B). On the first day of training, the reward was visible below the correct object for the first few trials (see methods), resulting in performance significantly above chance on day 1 across groups (*p* < 0.01 for all groups), therefore day 1 was excluded. The first day on which any group average reached ≥80% was day 12, with the young KD rats performing at an average of 80.20 ± 11.81% (Figures 4A,B). Thus, for additional diet and age comparisons, all analyses were focused on days 12–15. Prior to day 12, it is likely that a majority of animals were still acquiring the procedural aspects of the behavior and had not learned the object-in-place rule. A RM-ANOVA across days 12–15 revealed a significant main effect of day [*F*_(3,105)_ = 4.41; *p* < 0.01] on percent correct object choices, indicating that rats successfully acquired the rule during this phase of testing (Figure 4C). There were significant main effects of both diet [*F*_(1,35)_ = 4.25; *p* = 0.047] and age [*F*_(1,35)_ = 5.39; *p* = 0.03], but there was no significant interaction between age and diet [*F*_(1,35)_ = 0.69; *p* = 0.41]. Moreover, there was no effect of either age [*F*_(1,35)_ = 0.04; *p* = 0.84] or diet [*F*_(1,35)_ = 2.58; *p* = 0.12] on the turn bias indices during this time period (Figure 4D), indicating rats across all groups were still alternating throughout WM/BAT testing.

To ensure that rats were not adopting an alternative strategy rather than using the object-in-place rule (e.g., a non-match to sample or simple object alternation strategy) to solve the BAT, rats were tested for 1 day on a random arm version of this task within the elevated figure-8 maze. During this task, rats were forced to enter a particular arm pseudorandomly throughout the 20 trials. A two-factor ANOVA revealed no effect of age [*F*_(1,35)_ = 1.20; *p* = 0.28] or diet [*F*_(1,35)_ = 0.57; *p* = 0.46] on performance on random arm BAT (Figure 4E). This is likely because all rats had acquired the object-in-place rule and this strategy was being similarly used by all age and diet groups. Furthermore, performance on the random arm version did not significantly differ from performance on the final day of WM/BAT testing [*F*_(1,35)_ = 1.55; *p* = 0.22], nor was there a main effect of age [*F*_(1,35)_ = 2.20; *p* = 0.15], or diet [*F*_(1,35)_ = 3.22; *p* = 0.08] or interaction between age and diet [*F*_(1,35)_ = 0.50; *p* = 0.49].

### KD-Fed Rats Demonstrate Significant Changes in Metabolism- and Signaling-Related Protein Expression Relative to CD-Fed Rats

**FIGURE 5:**
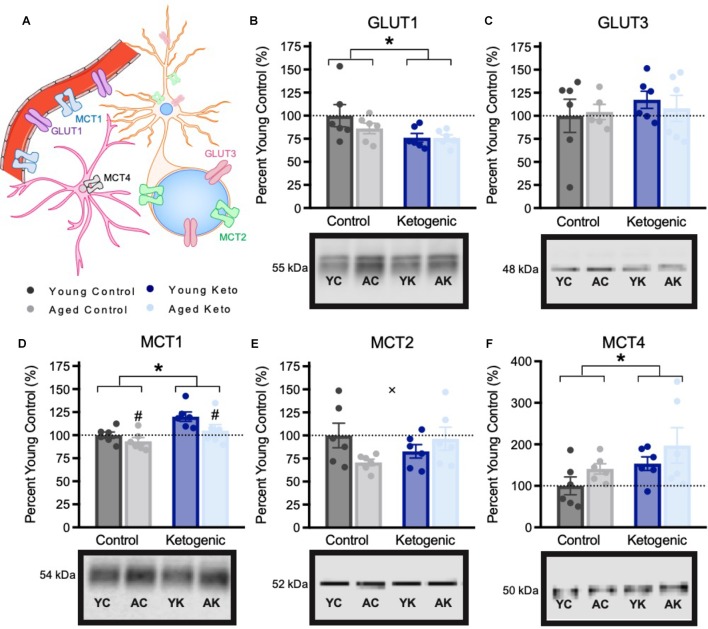
Metabolic transporter expression within the medial prefrontal cortex, shown as percent of young control rat expression (dotted line), with representative bands from each gel. **(A)** Schematic of locations of metabolic transporters within the central nervous system. **(B–C)** Expression of glucose transporters. **(D–F)** Expression of monocarboxylate transporters. All values are group means expressed as percent of young controls (dotted line) ± SEM, ^∗^indicates main effect of diet, #indicates a main effect of age and × indicates significant interaction between age and diet.

Due to the significant effect of the KD diet on behaviors that require both HPC and PFC, we quantified the expression levels of proteins in the PFC that were previously shown to be affected in the HPC by the KD (Hernandez et al., 2018a). Glucose and monocarboxylate transporter expression within the PFC was quantified in 6 rats per age and diet group (Figure 5). There was a significant decrease in expression of the GLUT1 transporter, which is found on epithelial cells of the blood brain barrier and some glial cells, within the PFC of rats in the KD group [*F*_(3,20)_ = 5.64; *p* = 0.03]. However, there was no effect of age [*F*_(3,20)_ = 0.94; *p* = 0.34] nor was there an interaction between age and diet [*F*_(3,20)_ = 0.87; *p* = 0.36]. For the GLUT3 transporter, which is expressed within neurons, there was no effect of age [*F*_(3,19)_ = 0.05; *p* = 0.83], diet [*F*_(3,19)_ = 0.12; *p* = 0.73], or significant interaction between age and diet [*F*_(3,19)_ = 0.24; *p* = 0.63]. MCT1, which is found on epithelial cells on the blood brain barrier, as well as some glial cells, had significantly decreased expression with age [*F*_(3,20)_ = 4.89; *p* = 0.04], and significantly increased expression in rats on the KD [*F*_(3,20)_ = 10.35; *p* < 0.01]. There was no interaction between age and diet [*F*_(3,20)_ = 0.75; *p* = 0.40] on MCT1 expression. MCT2, which is found on the cell bodies and post-synaptic terminals of neurons, also showed no main effect of age [*F*_(3,20)_ = 0.61; *p* = 0.45] or diet [*F*_(3,20)_ = 0.18; *p* = 0.68]. Interestingly, for MCT2 there was a significant interaction effect between age and diet [*F*_(3,20)_ = 4.58; *p* = 0.045]. Specifically, aged CD rats showed reduced MCT2 expression, whereas aged KD rats exhibited MCT2 levels comparable to those of the young CD rats. MCT4, which is located on astrocytes, had a significant main effect of diet [*F*_(3,20)_ = 4.45; *p* = 0.048] such that rats on the KD had increased transporter levels, but was not significantly affected by age [*F*_(3,20)_ = 2.66; *p* = 0.12], nor was the age by diet interaction significant [*F*_(3,20)_ < 0.01; *p* = 0.96].

**FIGURE 6:**
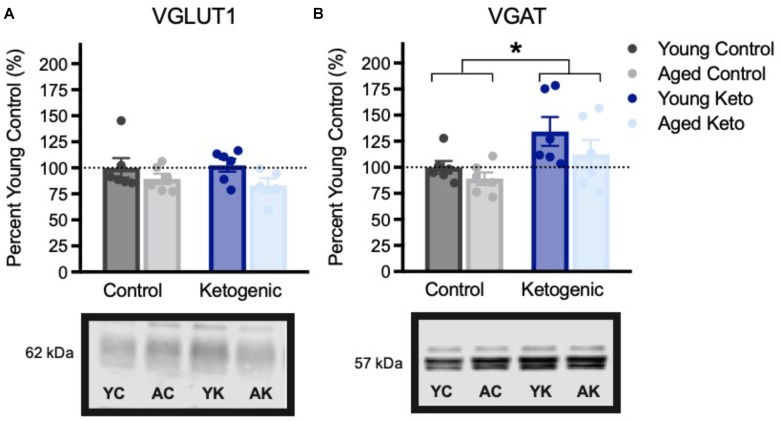
Vesicular transporter expression within the medial prefrontal cortex, shown as percent of young control rat expression (dotted line), with representative bands from each gel. **(A)** Vesicular glutamate transporter (VGLUT1) expression did not vary across age (*p* = 0.11) or diet (*p* = 0.90) groups. **(B)** Vesicular GABA transporter (VGAT) expression was significantly higher in rats in the KD-fed group relative to the CD-fed group, regardless of age (*p* = 0.01). All values are group means expressed as percent of young controls (dotted line) ± SEM, ^∗^indicates significant main effect of diet.

Expression of vesicular transporters for glutamate and GABA expression within the PFC were also examined (Figure 6). There were no significant differences across age [*F*_(3,20)_ = 2.75; *p* = 0.11] or diet groups in VGLUT1 expression [*F*_(3,20)_ = 0.02; *p* = 0.90], nor was there an interaction between the two variables [*F*_(3,20)_ = 0.04; *p* = 0.84]. This is distinct from the previous pattern observed in HPC in which aged control animals had reduced VGLUT1 expression, and this decline was reversed by the KD. There was, however, a significant increase in PFC VGAT expression in rats on the KD [*F*_(3,20)_ = 7.30; *p* = 0.01]. The effects of age [*F*_(3,20)_ = 2.38; *p* = 0.14] and the interaction between age and diet [*F*_(3,20)_ = 0.27; *p* = 0.61] were not significant. The increase in PFC VGAT expression in animals on a KD is similar to what has been observed in the HPC and suggests that the KD may enhance cortical inhibition. Critically, higher levels of GABA receptors in PFC are associated with better cognitive flexibility in rats (Beas et al., 2016) and increased GABA levels within PFC has been shown to correlate with better cognitive performance humans (Porges et al., 2017).

## Discussion

The current study confirmed our previous observation that across several months of consuming a high-fat, low-carbohydrate KD, aged rats were able to adapt and maintain a state of nutritional ketosis. This was evidenced by lower blood glucose levels and elevated levels of BHB in KD-fed rats compared to animals on a CD. Moreover, the significant increase in MCT expression and decrease in GLUT1 expression in the KD group provides supporting evidence that rats eating the high fat diet were utilizing ketone bodies, rather than glucose, as a primary fuel source. The novel findings from these experiments are that the consumption of a KD improved behavior on both the elevated figure-8 maze alternation task and a cognitive dual task that required working memory while simultaneously performing a bi-conditional association task (WM/BAT). Furthermore, rats on a KD demonstrated changes in metabolic- and vesicular transporter-related protein expression within the medial PFC that was dissociable from the changes previously reported for HPC (HPC; Hernandez et al., 2018a). Together these data have important implications for improving cognitive function in advanced age by targeting neural metabolism.

### The Ketogenic Diet and Cognitive Function

Although rats tend to default to an alternation strategy in most mazes (Lalonde, 2002; Hughes, 2004), because the right arm of the maze utilized in this study was enclosed by walls and the left arm of the maze was open (no walls; see Figure 1), rats required extensive training to alternate without a right-side bias. This pattern is typical avoidant behavior observed in rodents given the choice between open and closed arms (Pellow and File, 1986; Rodgers and Dalvi, 1997; Bailey and Crawley, 2009). The fact that rats on a KD were more willing to alternate correctly within an asymmetrical maze with one open arm indicates that the diet may be able to confer resilience in the face of an anxiogenic stimulus. Although the mechanism by which KDs can alleviate anxiety symptoms in humans or anxiety-like behaviors in rodents remains unknown, one potential mechanism is by altering GABAergic signaling (Yudkoff et al., 2001; Hernandez et al., 2018a), as the GABAergic system is known to play a role in anxiety regulation (Dias et al., 2013). This GABA mechanism may also interact with adenosine receptors to alter anxiety. Adenosine receptor knockout mice show enhanced levels of anxiety (Johansson et al., 2001; Giménez-Llort et al., 2002), and previous reports indicate changes in adenosinergic systems following KD administration (Masino et al., 2011). Furthermore, our data are in agreement with a previous study in which a ketone supplemented diet increased the amount of time rodents spent in the open arm of a an elevated plus maze (Ari et al., 2016), an effect modulated by blockade of adenosine receptors (Kovács et al., 2018).

Previous studies on the effect of a KD on cognitive function have been equivocal. One study reports decreased performance on the Morris watermaze following KDs (Zhao et al., 2004). These data, however, can largely be accounted for by variations in dietary implementation and composition when caloric intake is not monitored and KD-fed rats overconsume. Another study utilizing mouse models of Alzheimer’s disease reported no difference on the watermaze between KD-fed and control mice, but motor performance was better in KD-fed animals (Brownlow et al., 2013). Additionally, in a kindling model of seizure disorder, KD-fed and control rats performed similarly on the watermaze (Hori et al., 1997). Finally, it has been reported that there is no difference in fear conditioning between young rats on a KD or CD (Thio et al., 2010). The lack of improvement in some of these studies may have been due to near ceiling level performances by the young control animals. In contrast, a more recent study that compared a KD to exogenous ketone supplementation found that while both dietary manipulations improved watermaze performance, only the traditional KD improved novel object recognition (Brownlow et al., 2017). Other studies have also reported similar increases in performance on novel object recognition tasks in old mice on either traditional (Roberts et al., 2017) or cyclic KDs (Newman et al., 2017).

A key novel finding of this study is that all KD-fed rats demonstrated a significantly improved ability to correctly choose an object paired with its correct location. A random arm version of this task was used to rule out the possibility of rats adopting an alternative strategy (for example simply alternating which object they chose on each trial or utilizing a non-match-to-sample rule following an incorrect choice; Figure 4E). A critical aspect of the current study that can account for apparent discrepancies with previously reported data, was the utilization of a sufficiently complex task so that ceiling effects would not obscure the detection of cognitive improvements. The WM/BAT used here often requires more than 2 weeks of training for young animals to learn the rule (Hernandez et al., 2015, 2018b). Moreover, the WM/BAT has been shown to be particularly sensitive to cognitive decline in aging, with age-associated behavioral changes being detected prior to observable decline in the Morris watermaze task (Hernandez et al., 2015). Thus, the current data corroborate previous findings that there is an age-dependent impairment in the ability to acquire the object-in-place rule, which may arise from a reduced ability of the HPC and PFC to cooperatively support behavior, but this deficit was mitigated by nutritional ketosis. Together these data suggest that KDs may be able to enhance cognitive function across the lifespan. Notably, it is not possible at this point to dissociate enhanced cognitive function from decreased anxiety, and the improved performance demonstrated here likely results from a combination of several complimentary mechanisms.

### Biochemical Effects of the Ketogenic Diet and Potential Mechanisms for Cognitive Improvement

Our previous work reported alterations in the expression of metabolism- and neuronal signaling-related transporters in the HPC. Interestingly, the patterns of expression of these proteins in the PFC following a KD was distinct from what we observed in HPC. While the reduction in GLUT1 expression in both young and aged animals on the KD mirrored the results in HPC, likely reflecting a decreased need for glycolysis signaling pathways, several differences were observed between the PFC and HPC. First, GLUT1 protein within the PFC did not differ by age as it did within the HPC. Furthermore, no changes were found in GLUT3 expression within the PFC across any age or diet groups, though it was reduced within the HPC of KD-fed rats (Hernandez et al., 2018a). Additionally, within PFC there were notable differences in expression levels of monocarboxylate transporters, which transport ketone bodies, pyruvate, and lactate across membranes. The KD significantly increased expression of MCT1 in PFC, which transports ketone bodies across the blood brain barrier, and MCT4, which is found on astrocytes. While the expression of MCT2 within the PFC was lower in aged relative to young control animals, this difference was mitigated in rats on the KD, as indicated by a significant interaction between age and diet. This effect of age and diet was not observed in HPC for MCT2 (Hernandez et al., 2018a), providing evidence of dissociable biochemical effects of KD on PFC and HPC. It should be noted that there were minor differences in how the tissue samples were collected across these two studies. Although tissue was from the same subjects, and all tissue was frozen in the same way on the day of sacrifice, the HPC tissue was isolated before freezing, as opposed to PFC tissue which was isolated after freezing.

While our previous report (Hernandez et al., 2018a) found age- and diet-related changes in vesicular glutamate transporter (VGLUT1) expression within the HPC, no such changes were observed within the PFC. The levels of VGAT within the PFC, however, closely resembled those of the HPC, with increased expression in KD-fed animals of both ages relative to CD rats. If increased expression of VGAT represents an indirect measure of inhibitory capacity, this finding has several important implications. First, the mechanism by which the KD prevents epileptiform activity remains largely unknown, but here we report increased VGAT expression within two distinct brain regions, potentially increasing the threshold of activation required to propagate seizure activity. Second, administration of the GABA(B) receptor agonist baclofen can improve cognitive flexibility in rats (Beas et al., 2016), indicating that potentiating tonic inhibition by increasing levels of GABA may be beneficial for PFC-dependent tasks (Porges et al., 2017). These effects may be specific to behavioral paradigms requiring the inhibition of unwanted behaviors, such as in set-shifting or the BAT used here. Although direct infusion of GABA agonists into the PFC can improve some behaviors and induce anxiolysis (Shah et al., 2004), this can also result in behavioral deficits (Lee and Solivan, 2008; Hernandez et al., 2017b).

While transporter protein expression alone could certainly affect cognitive outcomes, it is likely that these changes, along with the ability to bypass glucose metabolism deficits, work in conjunction with several other physiological changes that results from the long-term consumption of a KD. For example, KDs significantly increase the amount of bioavailable ATP (Devivo et al., 1978; Kim et al., 2010), and increase the size and efficiency of mitochondria in the brain (Bough et al., 2006; Milder et al., 2010). Moreover, several signaling cascades mimicking those observed following fasting and caloric restriction are activated by a KD, including activation of PGC1α, a transcription factor that upregulates fatty acid transport, fat oxidation and oxidative phosphorylation (Jager et al., 2007). Finally, several other ways in which KDs can affect neuronal signaling have been suggested by others, including the ability to open ATP-sensitive K++ channels to slow spontaneous neuronal firing (Ma et al., 2007; Tanner et al., 2011), which may be elevated in old age (Wilson, 2005; Yassa et al., 2010; Thomé et al., 2016), and the ability to inhibit AMPA receptor firing (Chang et al., 2016). While this is not an exhaustive list, several comprehensive reviews that discuss the potential neurobiological effects of KDs have been published (Hartman et al., 2007; Baranano and Hartman, 2008; Paoli et al., 2014).

The current data could also have important implications for the treatment of anxiety-related disorders. HPC-PFC circuitry is highly sensitive to stress (Godsil et al., 2013), and synchrony between the HPC and PFC is elevated during emotional processing related to fear and anxiety (Adhikari et al., 2010), like that experienced in the open arm of a maze. Furthermore, individuals with general anxiety disorder and post-traumatic stress disorder have decreased HPC and PFC volume (Karl et al., 2006), as well as aberrant activity in both of these regions during fear-relevant tasks (Liberzon and Sripada, 2007; Hartley and Phelps, 2010; Jovanovic and Ressler, 2010). In fact, direct manipulation of the GABAergic system is one of the most heavily investigated mechanisms for alleviating anxiety (Lydiard, 2003; Durant et al., 2009; Gauthier and Nuss, 2015). Furthermore, therapeutics targeting GABAeric systems are among the most commonly prescribed treatment options for anxiety-related disorders (Nemeroff, 2003; Pillay and Stein, 2007; Feltner et al., 2008; Gauthier and Nuss, 2015). Thus, increasing inhibitory capacity may be beneficial during anxiety-inducing behaviors and prevent associated decreases in cognitive function. In fact, our behavioral data support the hypothesis that GABAergic changes within the PFC and HPC may alleviate anxiety-induced behavioral deficits.

An outstanding question regarding the KD’s mechanisms of efficacy for improving cognition, is whether the diet acts by simply improving metabolic function or if the ketone bodies themselves have signaling properties that improve cognition. In support of the former, glucose directly infused into the HPC (Ragozzino et al., 1998; Canal et al., 2005), or systemically injected (McNay et al., 2000), has been shown to improve rats’ performances on spontaneous alternation tasks. Long-term glucose consumption, however, can exacerbate insulin resistance and metabolic syndrome. Thus, it is not a long-term solution for enhancing cognitive function in old age. Intranasal administration of insulin is another approach that has been used to improve age-associated impaired glucose signaling. This has been shown to enhance cognition in older adults with memory impairments (Reger et al., 2008). In rats, however, chronic intranasal insulin administration failed to improve behavior in old animals and lead to reductions in markers of neuronal integrity (Anderson et al., 2017). Moreover, enhancing insulin receptor sensitivity pharmacologically, however, has produced mixed results regarding benefits to cognitive function in old age. Some studies have reported improved cognition (Kenawy et al., 2017), while others indicate a greater risk of impairment (Moore et al., 2013; Anderson et al., 2017; Thangthaeng et al., 2017), and an exacerbation of Alzheimer’s disease-related pathology (Barini et al., 2016). These data suggest that enhancing glucose signaling alone does not promote cognitive resilience in older adults and animal models. In fact, age-related declines in glucose transporters within the brain (Ding et al., 2013; Hernandez et al., 2018a), suggest that targeting glycolysis may be insufficient to overcome metabolic deficiencies. Conversely, the KD bypasses the requirement of glucose for energy metabolism, as well as elevates the levels of BHB and acetoacetate. BHB consumption alone has been shown to improve cognitive functioning in older adults with memory impairments (Reger et al., 2004). Critically, BHB and acetoacetate (another ketone body that is elevated by dietary ketosis) have signaling properties that are beneficial for cell function that include suppression of oxidative stress (Shimazu et al., 2013; Zou et al., 2016, Board et al., 2017, Newman and Verdin, 2014). Additional experiments will be needed, however, to clarify the relative efficacy of pharmacologically enhancing insulin receptor sensitivity versus elevating circulating levels of BHB and acetoacetate to improve cognitive outcomes in older adults.

## Conclusion

Recent work has demonstrated the potential of KDs initiated in young adulthood, even when implemented cyclically, to extend midlife longevity in mice and improve cognition (Newman et al., 2017; Roberts et al., 2017). Importantly the data presented here show for the first time that a late-life KD intervention may also be beneficial for improving cognitive outcomes. In fact, the differences in protein expression across the PFC and HPC have considerable implications for the development of therapeutic strategies for age-related cognitive decline and the early stages of Alzheimer’s disease. Designing systemic pharmacological treatments for specific mechanisms of age-related dysfunction within a single brain region, will undoubtedly have off target effects that will obscure the ability to improve overall behavioral output. Treatment strategies should therefore be designed that can elicit region-specific restoration of neurobiological function. The KD used in the current study appears to satisfy this criterion, at least for the PFC and HPC. However, additional data that examine the interaction between KDs and more global measures of network function, such as resting state network architecture and white matter integrity, will be necessary to elucidate the interactions between neuronal metabolism and overall brain health.

## Author Contributions

AH helped to design the experiments, collected and analyzed data, and wrote the paper. CH, KC, LT, QF, BM, and JM helped to collect the data. JM, AM, and JB helped design the experiments and interpret the data. SB designed the experiments, advised on the data collection and analysis, interpreted the data and wrote the manuscript.

## Conflict of Interest Statement

The authors declare that the research was conducted in the absence of any commercial or financial relationships that could be construed as a potential conflict of interest.
